# Association of the *HLA* locus and *TNF* with type I autoimmune hepatitis susceptibility in New Zealand Caucasians

**DOI:** 10.1186/2193-1801-2-355

**Published:** 2013-07-30

**Authors:** Jing H Ngu, Mary C Wallace, Tony R Merriman, Richard B Gearry, Catherine AM Stedman, Rebecca L Roberts

**Affiliations:** Department of Medicine, University of Otago, PO Box 434, Christchurch, 8140 New Zealand; Department of Gastroenterology, Christchurch Hospital, Christchurch, New Zealand; Department of Surgical Sciences, Dunedin School of Medicine, PO Box 913, Dunedin, 9054 New Zealand; Department of Biochemistry, University of Otago, PO Box 56, Dunedin, 9054 New Zealand

**Keywords:** HLA-DRB*03:01, HLA-DRB*04:01, HLA-A1-B8-DR3, FAS, CTLA4, Severe fibrosis, ALT levels

## Abstract

**Purpose:**

The precise etiology of autoimmune hepatitis (AIH) remains unknown, although a number of genetic loci have been implicated in the susceptibility of type 1 AIH. The purpose of this study was to test for association of these loci with type 1 AIH in New Zealand Caucasians.

**Methods:**

77 AIH patients and 485 healthy controls were genotyped for the SNPs rs2187668 (HLA-DRB*03:01), rs660895 (HLA-DRB*04:01), rs3749971 (*HLA-A1-B8-DR3*), rs231775 (*CLTLA4*), rs1800629 (*TNF*), and rs1800682 (*FAS*) using predesigned TaqMan SNP genotyping assays. Chi square analysis was used to test for association of allele and genotype with overall AIH, and with severe fibrosis and ALT levels at 6 months.

**Results:**

Significant risk of AIH was conferred by the minor alleles of rs2187668 (OR = 2.45, 95% CI 1.65-3.61, p < 0.0001), rs3749971 (OR = 1.89, 95% CI 1.21-2.94, p = 0.004) and rs1800629 (OR = 2.06, 95% CI 1.41-3.01, p = 0.0001). Multivariate analysis showed that rs2187668 was independently associated with type 1 AIH susceptibility (OR = 2.40, 95% CI 1.46-3.93, p = 0.001). The C allele of *FAS* SNP rs1800682 was associated with increased risk of severe fibrosis at diagnosis (OR = 2.03, 95% CI 1.05-3.93, p = 0.035) and with incomplete normalization of ALT levels at 6 months post-diagnosis (OR = 3.94, 95% CI 1.62-9.54, p = 0.0015).

**Conclusions:**

This is the first population-based study to investigate genetic risk loci for type 1 AIH in New Zealand Caucasians. We report significant independent association of HLA-DRB1*03:01 with overall susceptibility to type 1 AIH, as well as *FAS* with a more aggressive disease phenotype.

## Background

Autoimmune hepatitis (AIH) is a chronic progressive inflammatory liver disease of unknown etiology. It has a presumed autoimmune basis to its pathogenesis that is likely to involve a complex interaction of genetic and environmental factors (Czaja et al. 2010). Identification of these factors can potentially provide novel targets for treatment, diagnosis and prevention. AIH can be classified into two types according to circulating auto antibodies present in the patients’ sera. Type 1 AIH is characterized by the presence of anti-nuclear antibodies (ANA) and/or anti smooth muscle antibodies (SMA), whereas type 2 AIH is defined by the presence of anti-liver-kidney microsome antibodies (LKM1) (Czaja et al. 1995).

Type 1 AIH constitutes the vast majority of the disease in Caucasian populations. Genetic predisposition in type 1 AIH has been linked especially to the human leukocyte antigen (HLA) class II haplotype *HLA-A1-B8,* and the alleles HLA-DRB1*03:01 and HLA-DRB1*04:01 in North America and Europe (Doherty et al. 1994; Strettell et al. 1997). However, distinct differences in the pattern of association between genetic susceptibility and type 1 AIH are seen in populations from various geographical regions. For example, type 1 AIH is associated with HLA-DRB1*04:05 in Japan and Argentina (Seki et al. 1992; Pando et al. 1999), with HLA-DRB1*13:01 in Brazil and Venezuela (Fainboim et al. 1994; Czaja et al. 2002; Fortes Mdel et al. 2007) and with HLA-DRB1*04:04 in Mexico (Vazquez-Garcia et al. 1998). Polymorphisms in genes outside the *HLA* locus have also been associated with type 1 AIH. An A to G base-exchange polymorphism in exon 1 of the cytotoxic T-lymphocyte antigen 4 (*CLTLA4*) is associated with increased incidence of AIH in white North American and northern European patients (Agarwal et al. 2000; Djilali-Saiah et al. 2001), but not in South American (Bittencourt et al. 2003) and Japanese patients (Umemura et al. 2008). In young white AIH patients, the tumor necrosis factor (TNF) polymorphism -308G > A is associated with a poorer response to corticosteroid therapy. A Fas gene promoter polymorphism was found to influence susceptibility to AIH and its progression (Hiraide et al. 2005; Agarwal et al. 2007), leading to a more aggressive disease with an early development of cirrhosis (Hiraide et al. 2005). Polymorphisms in the vitamin D receptor gene have also been associated with AIH in German and Chinese patients (Vogel et al. 2002; Fan et al. 2005).

In New Zealand, only 2% of the AIH cases have a positive LKM1 antibody, indicating that type 1 AIH is the predominant disease in this population (Ngu et al. 2010). However, genetic loci previously shown to alter susceptibility to type 1 AIH have yet to be investigated in the New Zealand population. Our study had two aims. The first aim was to test for the association of six candidate risk loci (HLA-A1-B8, HLA-DRB1*03:01, HLA-DRB1*04:01, CTLA4, FAS, and *TNF*) in a population-based AIH New Zealand Caucasian cohort. The second aim was to examine whether these loci are associated with specific AIH phenotypes such as severe liver fibrosis stage at diagnosis and response to initial immunosuppression.

## Patients and methods

### Study population

Cases were New Zealand Caucasian with type 1 AIH who resided in the geographically defined region of Canterbury between 1^st^ July 2011 and 30^th^ June 2012. The list of AIH patients in Canterbury was generated from the established population-based AIH database that was set up in 2006 which has recorded information on the demography, biochemistry, serology, histology, radiology, and treatment of these patients. The methods used to identify these patients were described in detail in our earlier studies (Ngu, Bechly et al. 2010; Ngu et al. 2012). In brief, every new diagnosis of AIH in Canterbury was recruited prospectively since 2007. Cases diagnosed before 2006 were identified retrospectively by detailed searching of all private and public gastroenterology records in Canterbury. Cases were included into the database if they had definite or probable AIH as determined using the revised original scoring system (Alvarez et al. 1999). Cases that had a positive anti-LKM antibody (type 2 AIH) were excluded from this study. Stages of fibrosis were evaluated using the Metavir scoring system, and severe liver fibrosis was defined as Metavir stage 3 and 4. Response to initial immunosuppression was defined as normal ALT (<30 U/L) at 6 months from diagnosis, as incomplete normalization of ALT had been identified as an independent predictor of poor outcome in our earlier study (Ngu et al. 2013). Controls were healthy New Zealand Caucasians who were aged over 17 years of age at the time of the study and who had no personal or family history of autoimmune or inflammatory disease (Simkins et al. 2005). All study participants gave their informed written consent and ethical approval for this study was obtained from the Upper South A Regional Ethics Committee of New Zealand (approval number URA/10/07/055).

### Genotyping of susceptibility loci

Genomic DNA was obtained from peripheral blood using sodium chloride extraction (Miller et al. 1988) and re-suspended in Tris-EDTA buffer (pH 8.0) and stored at −20°C until analysis. Study participants were genotyped for all six loci using predesigned TaqMan SNP genotyping assays following the manufacturer’s instructions (Applied Biosystems, Foster City, CA, USA). Briefly, assays were performed in a total reaction volume of 5μl containing 2.5μl of 2× TaqMan^®^ Universal Master Mix (Roche Molecular Systems Inc, Branchburg, NJ, USA), 0.25 μl of 20× working mix of SNP genotyping assay and 12 ng of genomic DNA. The PCR was performed on an Roche LightCycler 480 Real-time PCR system, with an activation step of 10 min at 95°C, following by 40 cycles of denaturation (15 sec at 92°C) and annealing/extension (1 min at 60°C). Genotypes were assigned using the end-point genotyping analysis software. *CTLA4* rs231775, *FAS* rs1800682, and *TNF* rs1800629 genotyped using the predesigned TaqMan SNP genotyping assays C_2415786_20, C_9578811_10, and C_7514879_10, respectively. HLA-A1-B8, HLA-DR1*03:01, and HLA-DR1*04:01 were genotyped indirectly using the tagging SNPs rs3749971 (C_25983472_20) (Santos et al. 2008), rs2187668 (C_58662585_10) (Stanescu et al. 2011), rs660895 (C_26546458_30) (Ahmed et al. 2012). The web-based analysis programme SHEsis (http://analysis2.bio-x.cn/myAnalysis.php) (Shi et al. 2005) was used to check for deviations in Hardy-Weinberg Equilibrium (HWE).

### Statistical analysis

Tests for association of allele and genotype with overall AIH, and comparisons between groups with and without severe fibrosis at AIH diagnosis, and between those who did or did not normalize ALT within the first six months were made using chi square analysis and summarized as odd ratios (OR) with 95% confidence intervals (CI). Associations were considered significant if p < 0.05. Bonferroni corrections of α = 0.05/6 (p < 0.008) and α = 0.05/12 (p < 0.004) were used to adjust for multiple testing for associations with overall susceptibility and clinical phenotypes, respectively. Multivariate analysis, using a conditional logistic regression was used to determine the independent association of significant risk alleles identified from the univariate analyses.

## Results

### Characteristics of the study cohorts

Of the 85 type 1 AIH patients that fulfilled the study inclusion criteria, 77 cases (91% of the eligible cases) agreed to participate in this study. The basic demographics of these cases are shown in Table 1. All cases and controls (n = 455) were successfully genotyped for the six SNPs. No deviations from HWE were observed for any of these SNPs in either cases or controls.

**Table 1:** **Baseline characteristics of type 1 AIH patients at diagnosis**

Baseline features	
Total number of included cases	77
Female	61 (79%)
Male	16 (21%)
Caucasian	77 (100%)
Mean age (years)	45
Median age (years, range)	50 (12–74)
ALT U/L (mean, 95% CI)	571 (428–714)
Bilirubin umol/L (mean, 95% CI)	95 (62–128)
Albumin g/L (mean, 95% CI)	37 (36–38)
No. cases with histological stage ≥ Metavir 3	45 (58%)

### Association of *HLA, CTLA4, FAS* and *TNF* with type 1 AIH susceptibility

Chi-square analysis identified significant association of rs2187668 (HLA-DRB1*03:01), rs3749971 (*HLA-A1-B8-DR3*), and rs1800629 (*TNF)* with overall susceptibility to type 1 AIH (Table 2). No evidence of association of rs660895 (HLA-DRB*04:01), rs231775 (*CLTLA4*), or rs1800682 (*FAS*) with overall AIH susceptibility was detected. Minor allele A of HLA-DRB1*03:01 SNP rs2187668 conferred nearly 2.5 times increased risk of susceptibility to type 1 AIH (OR = 2.45, 95% CI 1.65-3.61, p < 0.0001). Minor alleles of both rs3749971 (*HLA-A1-B8-DR3*) and rs1800629 (*TNF*) were associated with 2 times increased risk of type 1 AIH with OR of 1.89 (95% CI 1.21-2.94, p = 0.004) and 2.06 (95% CI 1.41-3.01, p = 0.0001) respectively. All three associations remained significant after adjustment for multiple testing.

**Table 2:** **Allele and genotype frequencies of*HLA, CTLA4, FAS*and*TNF*SNPs in New Zealand Caucasian controls and patients with type 1 AIH**

SNP	Phenotype	Genotype frequency			Minor allele frequency	Unadjusted P (allele)	Odd ratio (allele) [95% CI]
rs2187668 (HLA-DRB1*03:01)		AA	AG	GG	A		
	AIH	10(0.130)	26(0.338)	41(0.532)	46(0.299)	<0.0001^±^	2.45 [1.65-3.61]
	Controls	13(0.029)	109(0.240)	333(0.732)	135(0.148)		
rs660895 (HLA-DRB1*04:01)		AA	AG	GG	G		
	AIH	41(0.532)	31(0.403)	5(0.065)	41(0.266)	0.10	0.72 [0.49-1.07]
	Controls	288(0.633)	145(0.319)	22(0.048)	189(0.208)		
rs3749971 (*HLA-A1-B8-DR3*)		AA	AG	GG	A		
	AIH	4(0.052)	23(0.299)	50(0.649)	31(0.201)	0.004^±^	1.89 [1.21-2.94]
	Controls	4(0.009)	98(0.218)	348(0.773)	106(0.118)		
rs231775 (*CTLA4*)		AA	AG	GG	G		
	AIH	33(0.429)	32(0.416)	12(0.156)	56(0.364)	0.42	1.16 [0.81-1.65]
	Controls	168(0.369)	212(0.466)	75(0.165)	362(0.398)		
rs1800629 (*TNF*)		AA	AG	GG	A		
	AIH	10(0.130)	29(0.377)	38(0.494)	49(0.318)	0.0001^±^	2.06 [1.41-3.01]
	Controls	20(0.044)	128(0.281)	307(0.675)	168(0.185)		
rs1800682 (*FAS*)		CC	CT	TT	C		
	AIH	19(0.247)	35(0.455)	23(0.299)	73(0.474)	0.93	1.01 [0.72-1.43]
	Controls	107(0.235)	214(0.470)	134(0.295)	428(0.470)		

^±^Significant at α=0.05 after Bonferroni correction (0.05/6 tests, p < 0.008).

Multivariate logistic regression was performed using both forward and backward stepwise analysis, and including all factors significant (p < 0.05) from the univariate association. This analysis showed that only rs2187668 (HLA-DRB1*03:01) was independently associated with overall susceptibility to type 1 AIH (OR = 2.40 [95% CI 1.46-3.93], p = 0.001). SNP rs3749971 (*HLA-A1-B8-DR3*) and rs1800629 (*TNF*) did not show independent association under this multivariate model. Further scrutiny indicated that the presence of minor allele rs2187668 (HLA-DRB1*03:01) was in fact significantly associated with the presence of minor allele rs3749971 (*HLA-A1-B8-DR3*) and rs1800629 (*TNF*) (p < 0.001).

### Association of *HLA, CTLA4, FAS,* and *TNF* loci with severe liver fibrosis and normalization of ALT

We examined whether these loci are associated with specific AIH phenotypes such as severe liver fibrosis stage at diagnosis and normalization of ALT at 6 months post diagnosis (Table 3). At diagnosis, 58% of the cases had severe liver fibrosis (Metavir ≥ 3), and 68% of the cases achieved complete normalization of ALT at 6 months post diagnosis. Within case analysis showed that whilst the C allele of the *FAS* SNP rs1800682 was significantly associated with incomplete normalization of ALT at 6 months post-diagnosis (OR = 3.94, [95% CI 1.62-9.54], p = 0.0015), this allele also increased the risk of severe fibrosis at diagnosis (OR = 2.03, [95% CI 1.05-3.93], p = 0.035). The latter association did not remain significant after Bonferroni correction. In addition, no association of rs231775, rs2187668, rs660895, rs3749971, or rs1800629 with severe fibrosis at diagnosis or with incomplete normalization of ALT at 6 months post-diagnosis was detected.

**Table 3:** **Association of*HLA, CTLA, FAS,*and*TNF*loci with severe fibrosis and normalisation of ALT in type 1 AIH patients**

SNP			Genotype			MAF	Unadjusted^±^P value (allele)	OR (allele) [95% CI]
rs2187668 (HLA-DRB1*03:01)			AA	AG	GG	A		
	Fibrosis ≥ Metavir 3	Yes	4(0.089)	16(0.356)	25(0.556)	24(0.267)	0.46	0.76 [0.38-1.55]
	(at diagnosis)	No	5(0.161)	10(0.323)	16(0.516)	20(0.323)		
	Incomplete normalization of ALT	Yes	1(0.067)	6(0.400)	8(0.533)	8(0.267)	0.67	0.82 [0.34-2.01]
	(at 6 months post diagnosis)	No	9(0.145)	20(0.323)	33(0.532)	38(0.306)		
rs660895 (HLA-DRB1*04:01)			AA	AG	GG	G		
	Fibrosis ≥ Metavir 3	Yes	27(0.600)	15(0.333)	3(0.067)	21(0.233)	0.22	1.56 [0.76-3.22]
	(at diagnosis)	No	13(0.419)	16(0.516)	2(0.065)	20(0.323)		
	Incomplete normalization of ALT	Yes	9(0.600)	4(0.267)	2(0.133)	8(0.267)	0.96	1.00 [0.40-2.46]
	(at 6 months post diagnosis)	No	32(0.516)	27(0.435)	3(0.048)	33(0.266)		
rs3749971 *(HLA-A1-B8-DR3)*			AA	AG	GG	A		
	Fibrosis ≥ Metavir 3	Yes	3(0.067)	13(0.289)	29(0.644)	19(0.211)	0.26	1.24 [0.54-2.83]
	(at diagnosis)	No	1(0.032)	9(0.290)	21(0.677)	11(0.177)		
	Incomplete normalization of ALT	Yes	0(0.000)	6(0.400)	9(0.600)	6(0.200)	0.98	0.99[0.37-2.68]
	(at 6 months post diagnosis)	No	4(0.065)	17(0.274)	41(0.661)	25(0.202)		
rs231775 *(CTLA4)*			AA	AG	GG	G		
	Fibrosis ≥ Metavir 3	Yes	20(0.444)	15(0.333)	10(0.222)	35(0.389)	0.29	0.69 [0.35-1.38]
	(at diagnosis)	No	13(0.419)	17(0.548)	1(0.032)	19(0.306)		
	Incomplete normalization of ALT	Yes	7(0.467)	6(0.400)	2(0.133)	10(0.333)	0.70	1.18 [0.51-2.74]
	(at 6 months post diagnosis)	No	26(0.419)	26(0.419)	10(0.161)	46(0.371)		
rs1800629 *(TNF)*			AA	AG	GG	A		
	Fibrosis ≥ Metavir 3	Yes	5(0.111)	19(0.422)	21(0.467)	29(0.322)	0.68	1.16 [0.57-2.35]
	(at diagnosis)	No	4(0.129)	10(0.323)	17(0.548)	18(0.290)		
	Incomplete normalization of ALT	Yes	1(0.067)	7(0.467)	7(0.467)	9(0.300)	0.81	0.90 [0.38-2.14]
	(at 6 months post diagnosis)	No	9(0.145)	22(0.355)	31(0.500)	40(0.323)		
rs1800682 *(FAS)*			CC	CT	TT	C		
	Fibrosis ≥ Metavir 3	Yes	14(0.311)	21(0.467)	10(0.222)	49(0.544)	0.035	2.03 [1.05-3.93]
	(at diagnosis)	No	5(0.161)	13(0.419)	13(0.419)	23(0.371)		
	Incomplete normalization of ALT	Yes	9(0.600)	4(0.267)	2(0.133)	22(0.733)	0.0015^±^	3.94 [1.62-9.54]
	(at 6 months post diagnosis)	No	10(0.161)	31(0.500)	21(0.339)	51(0.411)		

^±^Significant after Bonferroni correction (0.05/12 tests, p < 0.004).

## Discussion

AIH is a multi-factorial disease that is believed to develop when a genetically susceptible individual is exposed to an environmental factor that triggers the loss of immune tolerance towards hepatocyte antigens. The number and nature of the genes that play a role in AIH development is, at present, poorly defined. To date, the strongest genetic associations for type 1 AIH have been observed with genes located in the major histocompatibility complex (MHC), although several non-MHC genes have also been implicated in type 1 disease including *CTLA4, FAS*, and *TNF*. Our study is the first to investigate type 1 AIH susceptibility loci in New Zealand Caucasians.

In our cohort, the most significant association was observed between the HLA-DRB1*03:01 tagging SNP rs2187668 and overall AIH susceptibility, with the minor allele of this SNP conferring a strong risk effect. This finding is consistent with previous reports in European and North American Caucasian populations which have found the HLA-DRB1*03:01 allele to be the principle genetic risk factor for type 1 AIH in these populations. The HLA-DRB1*04:01 allele has also associated, albeit to a lesser degree, with the type 1 AIH in European and North American Caucasians (Czaja 2008). In our cohort, we did not find an association of HLA-DRB1*04:01 with overall susceptibility, despite having 100% power to detect an effect size of 5.97 at α=0.05 (Strettell, Donaldson et al. 1997). We also found that the minor allele of SNP rs1800629, which tags the extended haplotype *HLA-A1-B8-DR8*, was significantly associated with overall disease risk (p = 0.004, OR = 1.89, 95% CI 1.21-2.94) in New Zealand Caucasians, consistent with findings of previous studies (Donaldson et al. 1991, Al-Chalabi et al. 2008).

In addition to the *HLA* locus, we tested SNPs within the *CTLA4, FAS* and *TNF* genes for association with type I AIH. TNF is a key cytokine in the inflammatory response and variations in expression of TNF have been implicated in many autoimmune diseases. There is evidence suggesting that the promoter polymorphism *TNF -308G > A* (rs1800629) increases expression of TNF, and an association of this polymorphism with type 1 AIH has been reported in a number of cohorts (Tang et al. 2012). In our cohort of New Zealand Caucasians, the minor allele *TNF -308A* conferred susceptibility to AIH (OR = 2.06, 95% CI [1.41-3.01], p = 0.0001). In contrast to overall susceptibility, we found no evidence of association of *TNF -308G > A* with either severe fibrosis at diagnosis or incomplete normalization of ALT at 6 months.

*CLTLA4* codes for a T cell surface molecule that plays a key role as a negative regulator of T cell responses by inducing T cell apoptosis on binding to B7 molecules on antigen-presenting cells. *CTLA4* 49G/G genotype changes the sequence of *CTLA4* (threonine to alanine) and leads to diminished inhibitory effects on T cell proliferation, and hence, hyperactivity of T cells (Kouki et al. 2000). Several studies have examined *CTLA4* 49A > G (rs231775) in AIH although nearly all them have failed to show a definitive association between rs231775 polymorphism and disease susceptibility (Agarwal, Czaja et al. 2000; Bittencourt et al. 2003; Fan et al. 2004; Schott et al. 2007; Umemura et al. 2008). Nevertheless, a recent combined analysis of these studies comprising 526 patients with type 1 AIH and 631 matched controls reported the *CLTLA4* 49A/A genotype conferred protection against type 1 AIH (OR = 0.66, 95% CI 0.50-0.86) (Miyake et al. 2011). In our cohort we detected no association of the exon 1 SNP *CLTLA4* 49A > G (rs231775) with overall disease susceptibility. However, we did find an increased risk of severe liver fibrosis at presentation in patients who were homozygous for minor allele of rs231775 (OR = 8.57, 95% CI [1.04-70.89], p = 0.046), although this association was not significant after correction for multiple testing.

FAS is a member of the TNF receptor superfamily of membrane-bound molecules. *In vitro* studies have shown that FAS plays a prominent role in the induction of hepatocyte apoptosis and tissue destruction in AIH (Fox et al. 2001). Moreover the promoter polymorphism *FAS* -670 T > C results in higher expression of FAS on the surface of activated T cells (Sun et al. 2005) and, although this polymorphism has not been associated with overall susceptibility to type 1 AIH, the minor allele *FAS -670C* was found to confer protection against early onset cirrhosis in Caucasian type 1 AIH patients (Agarwal, Czaja et al. 2007). In our study we found that whilst *FAS -670A > C* was not associated with overall disease susceptibility in our New Zealand Caucasian patients, the *FAS -670C* allele confers risk of severe fibrosis at diagnosis (OR = 2.03, 95% CI 1.05-3.93, p = 0.035). In addition, the *FAS -670C* allele was significantly associated with failure to achieve complete normalization of ALT at 6 months post-diagnosis compared to the *FAS -670 T* allele (OR = 3.94, 95% CI 1.62-9.54, p = 0.0015). Our earlier study had demonstrated that incomplete normalization of ALT at 6 months is an independent predictor of liver related death (Ngu, Gearry et al. 2013). These observations suggest that the presence of the *FAS -670C* allele is associated with a more aggressive disease phenotype.

Multivariate analysis showed that rs2187668 (HLA-DRB1*03:01) was the only independent risk allele significantly associated with the overall susceptibility to type 1 AIH. This result is of interest as we have shown for the first time that while *HLA-A1-B8-DR3* and *TNF* were associated with type 1 AIH susceptibility, they were also significantly associated with HLA-DRB1*03:01. Our study demonstrates that, under the multivariate model, HLA-DRB1*03:01 was the dominant risk allele.

The strength of the present study is its population-based nature leading to results that are representative of the spectrum of AIH, as opposed to clinic-based studies where patients with more severe phenotypes are often over-represented. The weakness of this study is its small study size, and therefore it may be underpowered in confirming weaker associations. Nevertheless, it is important to note that despite the relatively small sample size, the results presented in this study did show highly significant associations.

## Conclusions

Our study is the first population-based study to test for association of selected genetic loci with type 1 AIH and also the first study performed in New Zealand Caucasians. We report significant independent association of HLA-DRB1*03:01 with overall susceptibility to type 1 AIH. FAS, whilst not associated with type 1 AIH susceptibility, was significantly associated with a more aggressive disease phenotype. These findings are an important first step in increasing our understanding of the genetic basis of type 1 AIH, and may help guide the selection of candidates for future functional studies.
